# Physical Activity, Health Benefits, and Mortality Risk

**DOI:** 10.5402/2012/718789

**Published:** 2012-10-30

**Authors:** Peter Kokkinos

**Affiliations:** ^1^Cardiology Department, Veterans Affairs Medical Center, 50 Irving Street NW, Washington, DC 20422, USA; ^2^Division of Cardiology, Department of Medicine, Georgetown University, 4000 Reservoir Road NW, Washington, DC 20057-2197, USA; ^3^Physical Therapy and Health Care Services, George Washington University, 2121 I Street, Washington, DC 20052, USA

## Abstract

A plethora of epidemiologic evidence from large studies supports unequivocally an inverse, independent, and graded association between volume of physical activity, health, and cardiovascular and overall mortality. This association is evident in apparently healthy individuals, patients with hypertension, type 2 diabetes mellitus, and cardiovascular disease, regardless of body weight. Moreover, the degree of risk associated with physical inactivity is similar to, and in some cases even stronger than, the more traditional cardiovascular risk factors. The exercise-induced health benefits are in part related to favorable modulations of cardiovascular risk factors observed by increased physical activity or structured exercise programs. Although the independent contribution of the exercise components, intensity, duration, and frequency to the reduction of mortality risk is not clear, it is well accepted that an exercise volume threshold defined at caloric expenditure of approximately 1,000 Kcal per week appears to be necessary for significant reduction in mortality risk. Further reductions in risk are observed with higher volumes of energy expenditure. Physical exertion is also associated with a relatively low and transient increase in risk for cardiac events. This risk is significantly higher for older and sedentary individuals. Therefore, such individuals should consult their physician prior to engaging in exercise.

“Walking is man’s best medicine”

Hippocrates

## 1. Introduction

Over 2,500 year ago, Hippocrates noted the potential health benefits of daily exercise of moderate intensity such as a simple walk. In the last six decades, and since the landmark work by Morris and coworkers [1], the plethora of epidemiologic evidence accumulated supports unequivocally an inverse, independent, and graded association between physical activity, health, and cardiovascular and overall mortality in apparently healthy individuals [2–22] and in patients with documented cardiovascular disease [9].

The exercise-induced health benefits are in part related to favorable modulations of cardiovascular risk factors that have been observed with increased physical activity patterns or structured exercise programs [10]. Most recently the discovery that skeletal muscles are capable of communicating with other tissues by the release of myokines into the circulation aids to our understanding of the exercise-induced health benefits on the molecular level. More specifically, Boström and colleagues [23] identified a new hormone irisin, named after the ancient Greek goddess of the rainbow and messenger of the Olympian gods. Irisin is released into the circulation by the skeletal muscle during exercise and triggers the transformation of white fat cells to cells that behave similar to brown fat cells (brown-in-white or brite cells). Moreover, mice engineered to express high irisin levels in blood were resistant to obesity and diabetes. These findings provide a mechanistic explanation for the protection exercise offers against metabolic diseases and perhaps a network of other chronic human diseases [23].

This paper examines the association between physical activity and mortality risk for apparently healthy populations and those with chronic health conditions.

## 2. Occupational and Leisure Time Physical Activity Studies

In a landmark study, Morris and coworkers reported that individuals with physically demanding occupations (London mail carriers and double-decker bus conductors) had approximately 50% lower rates of coronary heart disease (CHD) when compared to the more sedentary bus drivers and desk clerks [1]. These findings stimulated worldwide interest in the relationship between physical activity and cardiovascular mortality.

Subsequent leisure time physical activity studies and occupational studies from variety of industries included postal workers, railroad workers, and farm workers, employees of utility companies, civil servants, longshoremen, policemen, and firefighters examined the physical activity-mortality relationship. Despite the subjective nature of questionnaires utilized to quantify physical activity, the overall findings of these studies support a graded reduction in the risk of coronary heart disease and death with increased level of physical activity. This association was similar for men and women regardless of age or other confounding factors and was as robust as that of established risk factors such as smoking, hypercholesterolemia, and hypertension [24, 25].

Contradictory to the findings of the aforementioned studies are those reported in a Finnish study. The rate of coronary heart disease mortality was greater among lumberjacks compared to less active farmers of the same region [26]. However, these findings must be interpreted with caution. Although farmers were less active than lumberjacks, they were not sedentary. Thus, the study compared highly active individuals (lumberjacks) to somewhat less active (farmers). This along with the higher fat consumption and smoking rates among lumberjacks is likely to have attenuated the positive effects of physical activity in the lumberjacks and showed more favorable outcomes for the farmers.

Three landmark studies based on over 16,000 Harvard Alumni [19–21] provided the first evidence of several essential concepts regarding exercise, fitness, and health. First, the existence of an exercise threshold was identified, beyond which health benefits are realized. In this regard, an exercise intensity of about 5-6 METs and an exercise volume threshold somewhere between 1,000 to 2000 Kcal per week were identified for significant reduction in mortality risk. Second, physical activity necessary for health benefits is of moderate intensity and volume. Third, the investigators observed a risk associated with excessive exercise. Mortality risk tended to increase slightly in those expending more than 3,500 Kcal per week exercising [21]. This will be equivalent to about 30–35 miles of jogging per week. Forth, mortality risk was related to caloric expenditure regardless of the type of activity. When mortality risk was assessed based on different types of physical activity that included walking (miles/week), stair-climbing (floors), and sports paying, the inverse and graded association between mortality risk and volume of physical activity was again evident and in accord with their previous findings [19]. 

Finally, the exercise-related health benefits were only evident if physical activity was maintained throughout life. Those who played varsity sports, but did not maintain a physically active lifestyle had higher mortality rate compared to those who maintained a physically active lifestyle in adulthood. Moreover, those who avoided athletics in college but subsequently took up a more active lifestyle also had similarly low rates of mortality [20]. This finding suggests for the first time that the exercise-related health benefits can be attained at a later age in life. It also casts doubt that genetic factors that favor athletic abilities are also associated with better health and longevity. Yet, the influence of genetic factors in the reduction of the mortality risk cannot be dismissed. Furthermore, the argument can be made that it is not the physical activity that provides protection but the genetic composition of these individuals.

In this regard, the independent association of physical activity and mortality and the influence of genetic and other familial factors were assessed in a cohort of same-sex twins (7,925 men and 7,977 women). Individuals who reported engaging in brisk walking for a mean duration of 30 minutes, at least 6 times per month were classified as physically active. Those who reported no leisure time activity were classified as sedentary. The remaining individuals were classified as occasional exercisers. When compared to the sedentary twins, the adjusted risk of mortality was 33% lower among the twins who exercised occasionally and 44% lower among the physically active twins. The investigators concluded that physical activity is associated with lower mortality independent of genetic and other confounding factors [11].

## 3. Physical Activity and Mortality in Women

The findings of recent large cohort studies in women, including the Women's Health Study, the Lipid Research Clinics Research Prevalence Study, and the Women Take Heart Project also support an inverse and graded association between increased physical activity and mortality in women [7, 8, 13–15]. There is also evidence to support that physical activity may provide a greater degree of protection in women than men [13–15]. A noteworthy finding was that sedentary women who became physically active between a baseline and a follow-up visit (6 years apart) had 32% and 38% lower all-cause and cardiovascular mortality rates, respectively, compared with women who were sedentary at both visits [15]. This weakens the notion that the higher mortality reported in sedentary individuals is the consequence of subclinical diseases and not lack of physical activity per se (reverse causality).

In summary, the accumulated epidemiological findings support a strong, inverse, and graded relationship between volume of physical activity and all-cause mortality in men and women. The contribution of physical activity to health outcomes is independent even when the traditional cardiovascular risk markers and genetic factors are considered. Moreover, the degree of risk associated with physical inactivity is similar to and in some cases even stronger than the more traditional cardiovascular risk factors [2, 3, 9, 11–14].

An exercise volume threshold can be defined beyond which a significant reduction in mortality risk occurs. Such threshold appears to be at caloric expenditure of approximately 1,000 Kcal per week was defined as the threshold for an average reduction of 20% to 30% in mortality risk. Further reductions in risk are observed with higher volumes of energy expenditure. The independent contribution of the exercise components, intensity, duration, and frequency to the reduction of mortality risk is not clear and the need for more research to better understand the contribution of each component is emphasized [28].

Finally, it is important to emphasize that occupational and leisure time studies have used questionnaires to quantify the physical activity of the cohort. The assessment of physical activity with this method is subjective and the exercise volume reported is likely to be overestimated. Although this may be viewed as a weakness in these studies, the health benefits observed by these studies are likely to be underestimated.

## 4. Fitness Assessment Studies

More recent studies assessed fitness status by standardized exercise protocols. Such protocols are designed to progressively increase the workload until the age-predicted maximal heart rate is achieved or the participant can no longer continue. The aerobic workload achieved at this level, referred to as the peak exercise capacity, is expressed in metabolic equivalents (METs). One MET is equal to 3.5 mL of O_2_/kg/min, the amount of energy expended per kg of body weight, during one minute of rest. Any activity above resting requires greater oxygen consumption, and therefore, yields a higher MET level [29].

Because such exercise tests are designed to push the participant to exhaustion, fitness assessment by this method is far more objective than questionnaires. Select studies are presented here, the findings of which not only supported those of the occupational and leisure time studies, but more importantly, enriched our understanding of the association between exercise or increased physical activity and health benefits.

Three reports from the Aerobics Center Longitudinal Study (ACLS) are noteworthy [4–6]. In the first report Blair and coworkers reported a strong and inverse association between fitness levels and mortality in a large sample of mostly white, middle-aged, and relatively healthy individuals [4]. The largest reduction in mortality was observed between the least-fit and the next least-fit category (those achieving 6 to 8 metabolic equivalents (METs) versus those achieving <6 METs). Mortality risk continued to decrease with increased fitness and reached an asymptote at 9 and 10 METs for men and women, respectively [4]. The mortality risk associated with fitness was similar to that for cigarette smoking and elevated cholesterol levels [5]. When mortality risk was assessed in terms of change in exercise capacity between 2 visits (an average of 5 years apart) a 7.9% reduction in mortality was observed for every minute increase in peak treadmill exercise time between examinations. In addition, unfit men who improved their fitness status during subsequent visits had a 44% reduction in mortality risk [6]. We also reported similar finding in a cohort of older veterans who had repeated exercise tests over time (median 4 years). More specifically, when compared to the reference group (unfit in both visits) mortality risk was approximately 60% lower for individuals who were fit in both visits. For those who were unfit in the initial visit and improved their fitness status in subsequent visits mortality risk was 35% lower. For those who were fit initially, but their fitness declined in subsequent visits fit mortality risk was approximately 40% lower [30]. These findings support four concepts: (1) maintaining a relatively good fitness status over the years lowers mortality risk by approximately 60%; (2) improvements in fitness status at any age yields health benefits (35% lower risk); (3) decline in fitness status results in a concomitant attrition of the health benefits observed in those who maintained fitness over the years. However, the fitness-related health benefits persist to some degree even after fitness status declines. This and similar reports in women [16] weakens the notion that the higher mortality reported in sedentary individuals is the consequence of subclinical diseases and not lack of fitness per say (reverse causality).

Because exercise capacity can be quantified (peak METs) when standardized exercise testing protocols are utilized, several studies expressed this relationship in the context of survival benefit per MET increase in exercise capacity. The reduction in mortality risk per MET in these studies has ranged between 10% and 25%, for individuals with and without cardiovascular disease [3, 9, 16–18] and regardless of age, gender, or race [2, 3, 6, 9, 12–14, 16–18].

## 5. Association of Mortality Risk with Exercise Type, Duration, and Intensity

Evidence from several studies suggests an independent contribution of the exercise mode and each exercise components (intensity, duration, frequency) to mortality risk [28].

A great deal of information is available for the association between aerobic type of activities and mortality risk, while limited information exists for resistance or weight training. However, the relatively large Health Professionals' Follow-up Study (*n* = 44 452 men) provides the much needed information regarding resistance training and mortality risk association. Based on this study, the reduction in coronary heart disease risk achieved by participation in resistance training was similar to that provided by brisk walking and rowing, but was approximately half of that provided by running [22]. This, along with a much stronger association observed between exercise intensity and mortality risk suggest that exercise intensity may be more important than duration for lowering the risk of coronary heart disease.

In contrast to these findings [22], exercise duration was more effective in lowering the risk for coronary events than exercise intensity in the Women's Health Study [7, 8]. For the same exercise volume achieved by either higher exercise intensity or longer duration, the risk reduction was substantially greater for exercise duration. 

The potential for greater health outcomes associated with relatively high intensity-short duration exercise training have generated interest in programs utilizing high-intensity aerobic interval training. Such training consists of multiple short bouts of activity (usually 3 to 4 minutes in duration), followed by approximately equal periods of low-intensity activity. The intensity is usually >85% of peak oxygen uptake or peak heart rate. Several studies utilizing such exercise training have shown significant improvements in metabolic and cardiovascular parameters in both young healthy adults and heart failure patients. Compared with traditional continuous exercise programs, interval training has resulted in marked improvements in skeletal muscle capacity for fatty acid oxidation in young women [31] increases in muscle oxidative capacity in young men [32], and resting blood pressure reduction in middle-aged and older individuals [33].

Intuitively, high-intensity training raises safety concerns, especially in older individuals and those with heart disease. In this regard, a recent study performed in heart failure patients is noteworthy [34]. Elderly patients with stable postinfarction heart failure (ejection fraction < 40%) receiving optimal medical therapy were randomized to either a moderate continuous exercise program at 70% of peak heart rate, interval training at 95% of peak heart rate, or a control group. After 12 weeks, the high-intensity interval training resulted in greater improvements in aerobic capacity, mitochondrial function, and skeletal muscle flow-mediated dilation. High-intensity interval training induced reverse left ventricular remodeling and improved cardiac function. The program was also well tolerated, and quality of life improved.

Although these findings are impressive, it is imperative that they be interpreted with caution. Higher exercise intensities carry an inherent risk of serious complications, especially for those with compromised cardiac function and therefore the risk/benefit ratio may be unfavorable for certain individuals. Finally, it should be emphasized that the aforementioned findings are based on a few studies with relatively small number of participants. Larger studies are needed to confirm their findings.

However, it is important to keep in mind that the interaction between exercise intensity, duration, and frequency is inextricable. Higher exercise intensity may offer additional health benefits for some populations while others may benefit more from relatively low exercise intensities and longer duration. The salient message from these and other reports [12, 35] is that moderate activity such as brisk walking has a considerable health benefits and walking or similar activities a few hours per week should be recommended to all adults, as suggested in various guidelines [36, 37].

## 6. Physical Activity and Cardiac Risk Factors

### 6.1. Obesity

Obesity is defined as the accumulation of excess body fat usually ≥25% of the total body weight for men and ≥33% for women [38]. Because direct body fat assessment is impractical for large populations, body mass index (BMI) was adopted by The American Heart Association (AHA) as a practical clinical indicator of adiposity [39]. BMI, calculated as weight (kg) divided by height (m) squared (kg/m^2^), has been shown in large epidemiologic studies to correlate well with total body fat and to be related to cardiovascular and all-cause mortality [40–42]. 

The National Heart, Lung, and Blood Institute and World Health Organization have adapted a single set of cut-points at 5 BMI internals to classify overweight and obesity [38]. Based on this classification men and women with a BMI ≥ 30 kg/m^2^ are considered obese and at high risk for mortality (Table 1). However, it is important to mention that BMI, although practical, it can be misleading especially in athletes with relatively high lean body mass.

The mechanisms and causes of obesity are poorly understood. All experts agree that obesity is the result of chronic imbalance between caloric intake and caloric expenditure. Simply stated it, obesity is the result of chronic imbalance between energy intake in the form of food and drink, and total energy expenditure. However, why some people get fat while others don't, even when food intake is similar is poorly understood. This suggests that a chronic energy imbalance that favors weight gain may be the outcome of a complex interaction between genetic and environmental factors [43, 44].

 The predominant environmental factors for obesity appear to be overconsumption of calories and reduction in physical activity. Of the two, physical inactivity appears to play the predominant role. According to the USA federal report on obesity, total caloric intake over the last two decades has not substantially increased while physical activity has decreased significantly [45].

## 7. Physical Activity, Fitness, Obesity, and Mortality

Obesity and overweight are considered to be leading risk factors for a number of chronic health conditions, including diabetes mellitus, hypertension, coronary heart disease, and premature mortality. Obesity not only increases coronary heart disease risk directly, but also enhances it indirectly through its adverse effects on several established risk factors, including insulin resistance and hypertension.

Interventional studies show relatively modest weight reductions achieved with structured programs of physical activity. However, in a recent trial, cardiac rehabilitation patients randomized to an intensive counseling, and exercise program designed to achieve an energy expenditure of 3000 to 3500 kcal/wk or standard cardiac rehabilitation group, experienced double the weight loss (8.2 ± 4 versus 3.7 ± 5 kg) [46].

Despite the relatively low exercise-induced weight reduction, findings from large epidemiological studies support the concept that reduced risk mortality occurs among more active individuals regardless of body weight [47]. In a large follow-up study of 25 714 men, higher fitness levels were associated with lower risk of mortality in normal-weight, overweight, and obese men [48]. In addition, the higher mortality risk associated with higher waist circumference was trivialized after adjustment for fitness [49]. These investigators suggested that it is as important for clinicians to assess the fitness status of an overweight or obese patient as it is to evaluate blood pressure, inquire about smoking habits, and measure fasting plasma glucose and lipid levels.

Similarly, in two reports from the Nurses' Health Study (*n* = 204 957 combined), after adjustment for age, smoking status, parental history of coronary heart disease, menopause, hormonal use, and alcohol consumption, higher levels of physical activity in women were associated with reduced mortality risk across all categories of body weight [50, 51]. It is noteworthy that the mortality risk associated with obesity was attenuated by higher levels of physical activity, but was not totally eliminated. Similarly, being lean did not counteract the increased risk associated with being physically inactive [50, 51].

Other prospective studies performed over the last decade have assessed the independent and joint associations between body weight, fitness, physical activity patterns, and outcomes. The findings of these studies support that both physical inactivity and excess weight are independently associated with the increased risk of cardiovascular disease [52, 53]. However, a consistent finding of these studies is that fitness attenuated mortality risk regardless of body weight. When stratified within a given category of body dimensions (body mass index, waist circumference, or weight), subjects who are more physically active or fit consistently have a lower risk for adverse outcomes compared with those who are inactive or unfit.

Recent epidemiological findings have also drawn attention to an inverse association between body mass index and mortality in some populations, often termed the obesity paradox [54–56]. One explanation may be that individuals within the lowest body mass index category may have had undefined chronic illness, resulting in nonvolitional weight loss [55]. This is supported by two recent report from the Veterans Affairs database. In the first report, the inverse association between body mass index and mortality observed in male veterans was attenuated when fitness levels were considered suggesting that individuals with low body weight and low exercise capacity may have had undetected chronic disease [56, 57]. In the second report, weight gain over a mean follow-up period of seven years was related to lower mortality and weight loss was related to higher mortality when compared with stable weight [57].

## 8. Hypertension

Chronic hypertension is a major and most common risk factor for developing cardiovascular disease and mortality [58]. The mortality risk doubles for every 20-mm Hg increase in systolic blood pressure above the threshold of 115 mm Hg and for every 10-mm Hg increase above the diastolic blood pressure threshold of 75 mm Hg [59]. Approximately one third of the adult population in the United States has hypertension [60] and about 1 billion worldwide, with a 60% increase in the year 2025 [61]. The progressive increase in blood pressure and consequently, hypertension do not appear to be a fundamental feature of human aging [62], but the outcome of lifestyle factors such as diets high in fat, excess body weight and physical inactivity [61, 62]. Conversely, low-salt and/or low-fat diets, weight loss and increased physical activity, contribute significantly to blood pressure control [61, 63]. Increased fitness can also attenuate the rate of progression to hypertension [64].

## 9. Physical Activity, Fitness, and Hypertension

The significant impact of increased physical activity or structured exercise programs on blood pressure control has been consistently documented by a number of well-controlled studies. The general conclusion from these studies is that aerobic exercise training lowers blood pressure in individuals with stage 1 hypertension by 3.4 to 10.5 mm Hg for systolic blood pressure and 2.4 to 7.6 mm Hg for diastolic blood pressure [65–68]. The magnitude of the reduction may be related to the initial level of blood pressure [69] whereas the influences of age or gender are not clear [70].

Most of the work regarding exercise-related blood pressure changes has been in individuals with mild to moderate hypertension. Little is known on the effects of exercise in individuals with stage 2 hypertension. In this regard, we studied the effects of moderate-intensity aerobic exercise in male veterans with stage 2 hypertension and left ventricular hypertrophy. After 16 weeks of regular exercise we noted significant reductions in blood pressure and left ventricular mass regression in these patients. At 32 weeks, blood pressure in the exercise group was still significantly lower from baseline even after a 33% reduction in antihypertensive medication. There were no blood pressure changes in the no-exercise group [71].

Some studies suggest that low exercise intensity may be more effective in lowering blood pressure than high exercise intensity [69, 72, 73]. Others have noted that the favorable effects of training on blood pressure are not influenced or are only minimally influenced by exercise intensity, frequency, type, or duration [74]. Overall, the evidence that the blood pressure response to regular exercise differs according to training intensity is not convincing [65]. Information on the exercise-induced blood pressure changes over a 24-hour period (ambulatory blood pressure) is limited and blood pressure reduction is less dramatic than for blood pressure assessed from a single measurement (mean 3.0- and 3.2-mm Hg reductions for systolic and diastolic blood pressure, resp.) [66, 67]. However, two studies reported similar reductions of approximately 5–7 mm Hg for systolic and diastolic blood pressure [75, 76]. We also noted that daytime ambulatory blood pressure differences between fit and unfit prehypertensive men and women (*n* = 650) were similar to the blood pressure changes reported after exercise training [77]. In The HARVEST trial, young hypertensive patients who engaged in physical activity for at least once a week during the previous two months exhibited significantly lower 24-hour and daytime diastolic blood pressure, than the inactive group [78]. Others reported no significant reduction in blood pressure [79, 80]. However, in one of the two studies [80], only nine patients were included and no control group was used.

Almost all of the information regarding exercise and blood pressure is derived from aerobic exercises. Information available on the effects of resistance or strength training on resting blood pressure is limited and conflicting. The conclusion of a recent meta-analysis was that the average systolic blood pressure reduction as a result of resistance training was 3 mm Hg. This is substantially less than that reported for endurance exercise [81]. Based on these findings, the recommendation of the American College of Sports Medicine is that resistance training may serve as an adjunct to an aerobic-based exercise program for blood pressure reduction [67].

Despite the limited favorable changes in blood pressure, strength training is associated with numerous other health benefits, including reducing the risk of falls by reversing or attenuating the age-related decline in bone mineral density, muscle mass, and power [82]. Thus, according to the recommendations from the American Heart Association and the American College of Sports Medicine resistance exercise should be implemented as part of a complete exercise program [83, 84].

## 10. Exercise Blood Pressure Response

Evidence supports that the blood pressure response to submaximal workloads is associated with left ventricular hypertrophy (LVH). Middle-aged, prehypertensive individuals (*n* = 790) with similar resting blood pressure who achieved a systolic blood pressure ≥ 150 mm Hg at the exercise intensity of 4-5 METs had a significantly higher left ventricular mass index compared with those with systolic blood pressure below this level. Furthermore, the risk of LVH increased 4-fold for every 10-mm Hg rise in systolic blood pressure beyond the threshold of 150 mm Hg at 5 METs [85].

The rationale for the exercise systolic blood pressure and LV mass relationship is that the blood pressure response at exercise workloads of 4-5 METs reflects daytime blood pressure during most daily activities. Support for this contention is provided by the similarity between the exercise blood pressure at 5 METs and daytime ambulatory blood pressure obtained in 650 prehypertensive individuals [77, 85]. Thus, it is reasonable to assume that the daily exposure to relatively high blood pressure provides the impetus for an increase in LV mass even at the prehypertensive stage.

This exaggerated blood pressure response to exercise may be modulated by fitness. Exercise capacity is inversely related to exercise blood pressure and to LV mass [85]. We reported that the systolic blood pressure of fit individuals at exercise intensity of 4-5 METs and LV mass index were significantly lower when compared to the blood pressure and LV mass index of low-fit individuals. In addition, for every 1-MET increase in the workload achieved, we observed a 42% reduction in the risk for LVH [85]. Finally, 16 weeks of aerobic training resulted in significantly lower blood pressure at 3 and 5 METs [86] and regular exercise was associated with LV mass regression in older individuals with stage 2 hypertension [71]. Others have also reported significant reductions in blood pressure and regression in LV mass in older individuals with mild to moderate hypertension, after aerobic exercise [87].

## 11. Physical Activity, Fitness, and Mortality Risk in Individuals with Hypertension

**Figure 1:**
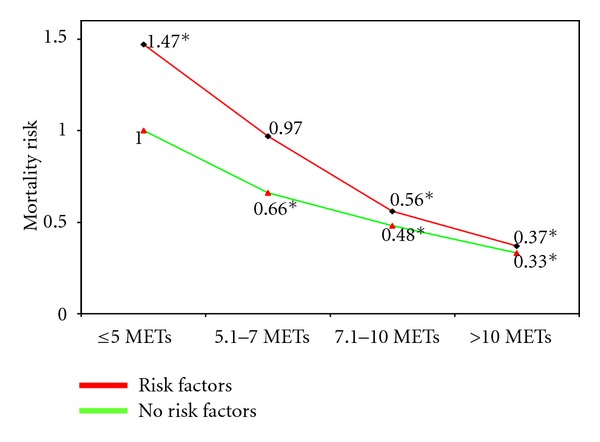
Mortality risk according to exercise capacity in hypertensive individuals with and without risk factors. Adopted from [27].

A number of large and well-controlled epidemiologic studies have reported an inverse and graded association between exercise capacity and mortality risk in hypertensive individuals [9, 27, 88]. In a cohort of 4,631 hypertensive veterans with multiple cardiovascular risk factors, we noted a 13% lower mortality for every 1-MET increase in exercise capacity. In addition, mortality risk was approximately 34% to 70% lower in those with an exercise capacity of >5 METs. When we examined the risk based on fitness status level (least fit to most fit) and on the presence or absence of additional risk factors, we observed the following. Among the least-fit individuals, those with additional risk factors had a 47% higher mortality rate than those without risk factors. The increased risk imposed by a low fitness level and additional cardiovascular risk factors was eliminated by relatively small increases in exercise capacity, and the risk declined progressively with higher exercise capacity [27] (Figure 1).

In a more recent study, we examined the mortality risk in hypertensive veterans according to fitness status and body mass index (BMI). Our findings support that the association between exercise capacity and mortality risk was strong, inverse and graded, in all BMI categories. For each 1-MET increase in exercise capacity the adjusted risk was 20% for normal-weight (BMI < 25); 12% for overweight (BMI: 25–29.9), and 25% for obese (BMI ≥ 35) [89].

To explore the fitness-fatness and mortality risk relationship further, we asked whether being of normal weight, but low fit carries a more favorable risk than being overweight or obese, but fit. Compared to the normal weight but unfit individuals, the mortality risk was 47% and 60% lower for the overweight-moderate-fit and overweight-high-fit individuals, respectively. Similarly, the risk was 55% lower for the obese-moderate-fit and 78% lower for the obese-high-fit individuals [89]. These findings suggest that it is more beneficial to be fit and overweight or obese rather than normal weight and unfit. Furthermore, it appears that obese hypertensive individuals may benefit at least as much from fitness than their overweight or normal weight counterparts.

## 12. Diabetes Mellitus

Evidence from large cohort studies supports that physical activity in general provides a highly effective way to delay or avert the development of diabetes mellitus in both men and women. The incidence of type 2 diabetes mellitus was inversely related to leisure time physical activity among men in the Harvard Alumni Study [90], in US male physicians [91] and women in the Nurses' Health Study [92]. The relative risk for developing diabetes mellitus in women was inversely related to the level of fitness as well as exercise volume and intensity in a dose-response fashion. Equivalent energy expenditures from different activities and intensities conferred similar health benefits.

These epidemiological findings are supported by two interventional studies. In the Finnish Diabetes Prevention Study [93], 522 middle-aged, overweight men and women were randomized to either an intervention group or a control group. In the Diabetes Prevention Program Research Group study, 3,234 nondiabetic individuals with elevated fasting glucose concentrations were randomly assigned to placebo, metformin, or lifestyle-modification program [94]. In both studies lifestyle interventions of a healthy diet, weight reduction and increased physical activity resulted in significantly lower cumulative incidence of diabetes mellitus than the control group [93, 94] and the group treated metformin [94]. Lifestyle interventions also resulted in more participants maintaining normal blood glucose values over a period of 3 years compared with the metformin or placebo group [94]. In addition, to prevent one case of diabetes during a period of three years, 6.9 persons would have to participate in the lifestyle-intervention program, and 13.9 would have to receive metformin [94].

## 13. Physical Activity, Fitness, and Mortality Risk in Individuals with Type 2 Diabetes Mellitus

Epidemiologic findings also support that increased physical activity is associated with lower mortality risk in individuals with type 2 diabetes mellitus. The all-cause mortality risk for low-fit or sedentary diabetics is more than 2 times higher compared with physically fit men and women diabetics regardless of body weight [95–97].

Low physical activity levels and poorer fitness status are inexorably associated with aging and age-related unfavorable changes in several physiological and metabolic processes, including declines in muscle mass, strength, endurance, and aerobic fitness, with reciprocal increases in adiposity, insulin resistance, metabolic syndrome, and type 2 diabetes mellitus [29, 98]. Individuals with diabetes mellitus, in addition, show an accelerated decline in oxidative capacity (maximum oxygen uptake), muscle mass, muscle strength, and glycemic control with aging [99–102]. Reduced exercise capacity is a strong predictor of all-cause mortality in older individuals with and without diabetes mellitus [49]. Since aging and reduced exercise capacity often coexist, and increased fitness is inversely related to mortality risk, we assessed the relationship between aging and exercise capacity in older individuals with diabetes mellitus. Our findings show that greater exercise capacity or fitness is associated with lower mortality risk in individuals aged 50 to 65 years old as well as those older than 65 with type 2 diabetes mellitus. When fitness categories were considered, the mortality risk was 30% to 80% lower for those who achieved more than 4 METs in both age groups.

Because poorer fitness and lower physical activity levels are strongly associated with aging and age-related unfavorable changes in several physiological and metabolic processes, it is imperative that healthcare providers encourage a physically active lifestyle regardless of age.

Because African-Americans have a 2- to 6-fold higher risk for developing diabetes mellitus [103] and approximately double the diabetes mellitus death rate [104] we probed for racial differences on the impact of exercise capacity on mortality risk in the Veterans Exercise Testing Study [105]. We found that exercise capacity was a strong predictor of mortality in black and white men with type 2 diabetes mellitus. The age-adjusted reduction was graded and more pronounced in whites than in blacks; each 1-MET increase in exercise capacity yielded 14% and a 19% lower risk for blacks and whites, respectively. Similarly, the risks were 34% and 46% lower for moderately and highly fit blacks versus blacks in the low-fitness category, respectively. For whites, the comparable reductions were 43% and 67%, respectively [105].

Recent evidence form large epidemiologic studies support significantly higher mortality risk only in individuals with relatively low body mass index (BMI) with no excess risk associated with overweight or obese individuals. This puzzling observation, termed obesity paradox [56, 106], has not been investigated in the context of fitness status in diabetic individuals. We therefore, assessed the interrelationship between exercise capacity, BMI and mortality risk, in middle-aged and older African-American and Caucasian veterans with type 2 diabetes mellitus [107, 108]. We observed a significantly higher mortality risk (70%) in those with BMI within the normal range (18.5–24.9 kg/m^2^) versus heavier subjects, consistent with the obesity-mortality risk paradoxical association [56, 106, 109]. When probing for racial differences, we observed that this paradoxical association was evident in both races, with a substantially higher mortality rate in African Americans (95%) than Caucasians (53%).

The lower mortality risk associated with increased fitness may be modulated by the exercise-related favorable effects on carbohydrate and fat metabolism. It is known that exercise is an insulin-independent stimulant of glucose uptake by the working muscle cells via the GLUT-4 transporter [110]. Exercise-induced translocation of GLUT-4 transporters is modulated by several factors related to muscular contractions such as increased calcium concentrations [111], hypoxia [112] and nitric oxide [113]. Exercise training studies support that both aerobic and anaerobic exercise training regimens improve glucose uptake and insulin sensitivity [114, 115].

The recent discovery of the hormone Irisin provide a mechanistic explanation for the protection exercise offers against metabolic diseases and perhaps a network of other chronic human diseases [23]. Irisin is released into the circulation by the skeletal muscle during exercise and triggers the transformation of white fat cells to cells that behave similar to brown fat cells (brown-in-white or brite cells). Moreover, mice engineered to express high irisin levels in blood were resistant to obesity and diabetes [23].

## 14. Physical Activity, Fitness, and Lipids

The most consistent findings from epidemiologic studies and randomized controlled trials supports that aerobic exercise of adequate intensity, duration, and volume results in favorable and independent alterations in high-density lipoprotein (HDL) cholesterol, with less consistency for reductions in total cholesterol, triglycerides, and low-density lipoprotein cholesterol concentrations for both normolipidemic and dyslipidemic individuals [116–118]. Exercise training can also attenuate the reductions in HDL cholesterol [119, 120] usually observed with low-fat diets [121, 122].

Overall, no consistent evidence is available to indicate that HDL cholesterol changes related to exercise are associated with age, ethnicity, or gender [118]. However, greater HDL cholesterol changes in men versus women, particularly when exercise is combined with a prudent diet, have been reported by some [119, 122], but disputed by others [123, 124]. Some studies suggested that exercise training does not appear to improve HDL cholesterol concentrations beyond the improvements seen with hormone replacement therapy [125], whereas others have shown synergistic effects between hormone replacement therapy and exercise training [126].

Epidemiological and interventional study evidence support that the magnitude of the changes in HDL cholesterol is related more to the volume of exercise than the intensity [116–118]. The exercise volume for achieving significant HDL cholesterol changes is estimated at 1000 to 1500 kcal/wk [127]. This parallels an earlier study in which a mean estimated weekly energy expenditure of 1245 kcal for individuals running 7 to 10 miles per week (average, 9 miles) and 1688 kcal for those running 11 to 14 miles per week (average, 12 miles) resulted in 7% and 11% increases in HDL cholesterol concentrations, respectively [128]. A dose-response relationship between the volume of exercise and HDL cholesterol changes has also been suggested by this study. For each mile increase in weekly distance, HDL cholesterol concentration increased 0.31-mg/dL. Compared with the sedentary group, HDL cholesterol concentrations were 7%, 11%, 12%, and 19% higher for individuals in the groups running average distances of 9, 12, 17, and 31 miles per week, respectively [128]. Similar findings were reported by others [128–131]. In women, menopausal status does not appear to influence this dose-response relationship [132].

## 15. Physical Activity, Fitness, and Inflammation

Although the mechanisms of atherosclerosis are not completely understood, it is well accepted that atherosclerosis is largely a chronic inflammatory disease of the arterial wall known as the “response-to-injury” theory [133–135]. This theory states that atherosclerosis develops as a result of repetitive injury and ongoing inflammatory process of the arterial endothelium, initiated by a pathogen. A number of blood markers have been identified that are associated with inflammation, most notably white blood cell count, C-reactive protein (CRP), homocysteine, fibrinogen, and other proteins involved in the immune response. The most widely studied inflammatory blood marker is CRP [136].

Several studies have shown that elevated levels of CRP are directly associated traditional cardiac risk factors and independently increase the risk of cardiovascular disease and mortality in both healthy individuals and patients with existing cardiovascular disease [137–141]. Acute exercise induces a transient inflammatory response, including heightened CRP concentration. This is most likely due to joint and muscle inflammation after vigorous activity. However, regular, sustained exercise has been shown to suppress inflammation. The findings of epidemiological studies consistently support of an inverse association between CRP, other inflammatory markers, and fitness. CRP levels of those engaging in regular exercise are approximately 30% to 50% lower when compared to sedentary individuals [142–144]. Collectively, these [142–144] and other findings [10] support that the health benefits (including lower mortality risk) associated with increased fitness may be explained in part by the inverse association between fitness and inflammatory markers.

## 16. Risk of Death during Physical Activity

The potential for death is inherent in structured exercise programs or physical exertion. The first death mentioned as a result of physical exertion is recorded by the Greeks. Legend has it that Phidippides ran from the battlefield of Marathon to Athens (approximately 26 miles away) to carry the news to the Athenians that they were victorious against the invading Persians. He reached Athens in perhaps 3 hours, delivered his message, and died shortly thereafter from exhaustion.

Was it the 26-mile run that killed Phidippides? Not likely! Less known is the fact that, only days prior to the battle at Marathon, Phidippides was sent to Sparta to ask for help. He ran the rugged, mountainous 140-mile course in about 36 hours to deliver the message. Afterwards, Phidippides ran that same 140-mile trail back to Athens with the disappointing news that the Spartans refused to send warriors to help the Athenians. A few days later he was in the battle of Marathon where, in all likelihood, Phidippides had been carrying messages back and forth to the different generals on the field during the day's battle. It was at the end of that last day when he was charged with running to Athens to deliver the victorious news [29].

Although we will never know exactly what killed Phidippides, the numerous modern marathon and ultramarathon races run by millions of runners annually are proof that humans are capable of such a task when trained properly. On the other hand, the occasional death of a runner reminds us of our vulnerabilities.

Almost all exercise-related deaths in previously asymptomatic adults without prior history of coronary heart disease have been the result of atherosclerotic plaque rupture in one of the coronary arteries that led to an acute coronary thrombosis [145–148]. Recent findings also suggest that demand ischemia (i.e., ischemia due to an imbalance between oxygen supply and demand) may be implicated in exercise-related acute coronary events during long-distance running [149].

Because the most common cause of cardiac complications is atherosclerotic coronary artery disease, the risk of exercise varies according to the population. In older populations where the prevalence of coronary atherosclerotic disease is high, the risk of death during exercise or physical exertion will be correspondingly high [150]. In apparently healthy, young individuals (<35 years), sudden death is often due to hypertrophic cardiomyopathy [150]. Interestingly, cardiac arrest, most commonly attributable to hypertrophic cardiomyopathy or atherosclerotic coronary disease, occurs primarily among male marathoners [149].

Several studies have also shown an inverse association between the risk of an event and the fitness status of the individual [151–154]. The risk of a cardiac event during physical exertion for sedentary individuals is reported to be approximately 2.5 to 30 times higher when compared to those who engage even moderate levels of physical activity (twice weekly) [152–154].

Despite this risk, the exercise-related cardiac event is relatively rare even in high-risk populations. The incidence of exercise-related deaths is the Rhode Island study [155] over a five-year period was 1 death per year for every 15,240 male joggers with no know coronary heart disease and 1 death per year for every 7,620 in those with known coronary heart disease. In general, the rate of sudden cardiac death and other cardiac events during exercise is estimated to be between 0 and 2 per 100,000 hours of exercise in the general population and 0.13 to 0.61 per 100,000 hours in cardiac rehabilitation programs [156, 157]. More recent evidence from a large cohort on marathon and half marathon runners concur with these findings. The incidence rates of cardiac arrest and sudden death during long-distance running races were 1 per 184,000 and 1 per 259,000 participants, respectively. This translates roughly into 0.2 cardiac arrests and 0.14 sudden deaths per 100,000 runner-hours at risk, using average running times of 4 and 2 hours for the marathon and half-marathon, respectively [149].

In conclusion, it is clear that physical exertion is associated with a transient increase in risk for cardiac events. This risk is significantly higher for older and sedentary individuals engaging in an exercise program. Therefore, it is prudent that such individuals consult their physician to evaluate the presence of subclinical coronary artery disease prior to engaging in an exercise program. 

## 17. Exercise Recommendations for Health Benefits

The plethora of information and (at time misinformation) regarding exercise, fitness, and health within the last few decades is stunning and overwhelming for most individuals. To minimize confusion for the patients, reduce the risk of injury, and to maintain exercise efficacy, health care providers must view exercise as an intervention similar to prescribing medication. The mode of exercise, its frequency, intensity and duration should be considered carefully. Moreover, exercise should be tailored to meet an individual's needs and abilities. This is especially true for special populations such as elderly, overweight or obese, hypertensive, and diabetic individuals. The current recommendations from the American Heart Association and the American College of Sports Medicine for middle-aged adults and older individuals [83, 84] stated below should be followed.

- Exercise should be primarily aerobic, supplemented by muscle-strengthening activities.
- The exercise intensity should be moderate (brisk walking at 15 to 20 minutes per mile) for most individuals and at even lower intensities for those unable to sustain such walking speeds. Moderate- and vigorous-intensity (jogging) activities can be combined for younger individuals or those able to sustain such intensities.
- The exercise duration should consist of ~30 minutes of continuous or accumulated physical activity on most, and preferably all, days of the week.
- A gradual increase in the minimum exercise volume is recommended to maximize health benefits. In addition, a minimum of 2 days per week of light weight-resistance exercises involving the major muscle groups and designed to maintain or increase muscular strength and endurance is encouraged.

## Figures and Tables

**Table 1:** Classification of body weight and obesity based on BMI and waist circumference.

	Obesity class	BMI (kg/m^2^)	Waist circumference	Associated health risk
Underweight		<18.5		
Normal		18.5–24.9		Average
Overweight		25.0–29.9	Men: ≥94 cm Women: ≥80 cm	Increased
Obesity	I	30.0–34.9	Men: <102 cm Women: <88 cm	Moderate
Moderate obesity	II	35.0–39.9		High
Severe obesity	III	≥40		Very high
